# Interaction of RNA-binding protein HuR and miR-466i regulates GM-CSF expression

**DOI:** 10.1038/s41598-017-17371-5

**Published:** 2017-12-08

**Authors:** Jing Chen, William Adamiak, Ganlei Huang, Ulus Atasoy, Abdolmohamad Rostami, Shiguang Yu

**Affiliations:** 1 Department of Neurology, Thomas Jefferson University, Philadelphia, PA 19107 USA; 2 Arkansas Biosciences Institute, Arkansas State University, Jonesboro, AR 72467 USA; 3 Department of Molecular Microbiology and Immunology, Department of Surgery, University of Missouri, Columbia, MO 65211 USA

## Abstract

Granulocyte-macrophage colony-stimulating factor (GM-CSF) produced by T helper 17 (Th17) cells plays an essential role in autoimmune diseases. Transcriptional regulation of Th17 cell differentiation has been extensively studied, but post-transcriptional regulation of Th17 cell differentiation has remained less well characterized. The RNA-binding protein HuR functions to promote the stability of target mRNAs via binding the AU-rich elements of the 3′ untranslated region (3′UTR) of numerous pro-inflammatory cytokines including IL-4, IL-13, IL-17 and TNF-α. However, whether HuR regulates GM-CSF expression in Th17 cells has not been fully investigated. Here we showed that HuR conditional knockout (KO) Th17 cells have decreased GM-CSF mRNA in comparison with wild-type (WT) Th17 cells, and that HuR binds directly to GM-CSF mRNA 3′UTR. Interestingly, HuR deficiency increased the levels of certain microRNA expression in Th17 cells; for example, miR-466i functioned to mediate GM-CSF and IL-17 mRNA decay, which was confirmed by *in vitro* luciferase assay. Furthermore, we found that HuR promoted Mxi1 expression to inhibit certain miRNA expression. Taken together, these findings indicate that interaction of HuR and miR-466i orchestrates GM-CSF expression in Th17 cells.

## Introduction

Naïve CD4^+^ T cell differentiate into different subsets of T helper cells after antigen stimulation that are crucial for orchestrating adaptive immune responses. Apart from the well characterized Th1 and Th2 cells that have been known for more than three decades ago, a new subset of Th17 cells has been identified in the past ten years^1–6^. Because of the essential and non-redundant role of Th17 cells in a number of autoimmune diseases including experimental autoimmune encephalomyelitis (EAE), an animal model of multiple sclerosis (MS), rheumatoid arthritis, and psoriasis^7–9^, regulation of Th17 cell differentiation has been extensively studied^10–13^. The signature cytokine for Th17 cells is interleukin 17 (IL-17); later researchers found that GM-CSF is also produced by Th17 cells^7,14^. Two research groups have independently demonstrated that GM-CSF is essential for Th17 cells to induce autoimmune neuroinflammation^7,14^. Further study showed that the responsiveness of bone marrow-derived monocytes to GM-CSF plays a pivotal role in EAE^6^. Thus, administration of recombinant GM-CSF resulted in more severe EAE^15^, and mice with transgenic overexpression of GM-CSF in T cells spontaneously developed autoimmune neuroinflammation^16^. Interestingly, elevated concentrations of GM-CSF have been reported in the cerebrospinal fluid of patients with relapsing-remitting MS^17,18^, suggesting that GM-CSF may play a similar pathogenic role in human MS. Thus, targeting GM-CSF by antibody has been tested in numerous clinical trials. Some trials have shown promising results in treatment of rheumatoid arthritis, and others are still in progress for treating MS patients^19^. A recent Phase I clinical trial showed that targeting human GM-CSF by MOR103 is safe^20^. Given the importance of Th17 cells and their cytokines in numerous types of autoimmune inflammation^7,10,14,21,22^, it is imperative to further study regulation of GM-CSF production in order to develop novel approaches for targeting it.

Gene expression is post-transcriptionally regulated by RNA-binding proteins (RBPs)^23,24^. Although most RBPs destabilize target mRNAs, a few of these, including HuR, bind to 3′UTR of proinflammatory cytokines to stabilize them^24–26^. In particular, HuR binds to adenylate-uridylate-rich elements (AREs) located in the 3′UTR of unstable genes to selectively mediate mRNA stabilization^23,27,28^. We previously showed that HuR post-transcriptionally stabilizes IL-17 and CCR6 mRNA and promotes their expression in autoimmune neuroinflammation^29,30^. However, it remains unclear whether HuR modulates GM-CSF expression in Th17 cells.

It is well known that interaction of HuR and microRNAs (miRNAs) controls target mRNA expression in response to environmental stimuli^31,32^. MiRNAs are small, non-coding RNAs with approximately 21 to 24 nucleotides (nt)^33^, which regulate the expression of numerous target genes by mediating their mRNA decay and/or repressing their translation^33–35^. The most common motif is perfect pairing between nucleotides 2 and 7 at the 5′ end of the miRNA, which is called the ‘seed’ region. It is thought that most miRNA-mRNA interactions involve the seed region at the 5′ end of miRNA. The small size of miRNAs provides a limited amount of information for specificity. Furthermore, as partial pairing between a miRNA and a target site is often sufficient for miRNA function, which means that a single miRNA can regulate multiple mRNAs but which also makes target predictions complicated^36^. Although cells contain hundreds of miRNAs, only a limited number of miRNAs have been validated functionally. Given the importance of miRNAs in regulating expression of numerous genes, further characterizing specific miRNA function will improve our understanding of gene regulation. Here we provide evidence that HuR post-transcriptionally modulates GM-CSF expression in Th17 cells. The level of miR-466i is increased in HuR KO Th17 cells compared with WT Th17 cells. The 3′UTRs of GM-CSF and IL-17 mRNA are potential targets of miR-466i, as shown by Targetscan analysis. *In vitro* luciferase transfection assay demonstrated that miR-466i could target GM-CSF and IL-17 mRNA 3′UTRs for decay, suggesting that miR-466i has potential as a novel reagent for therapeutic intervention in autoimmune inflammation.

## Results

### Knockout of HuR reduces GM-CSF expression in Th17 cells

**Figure 1:**
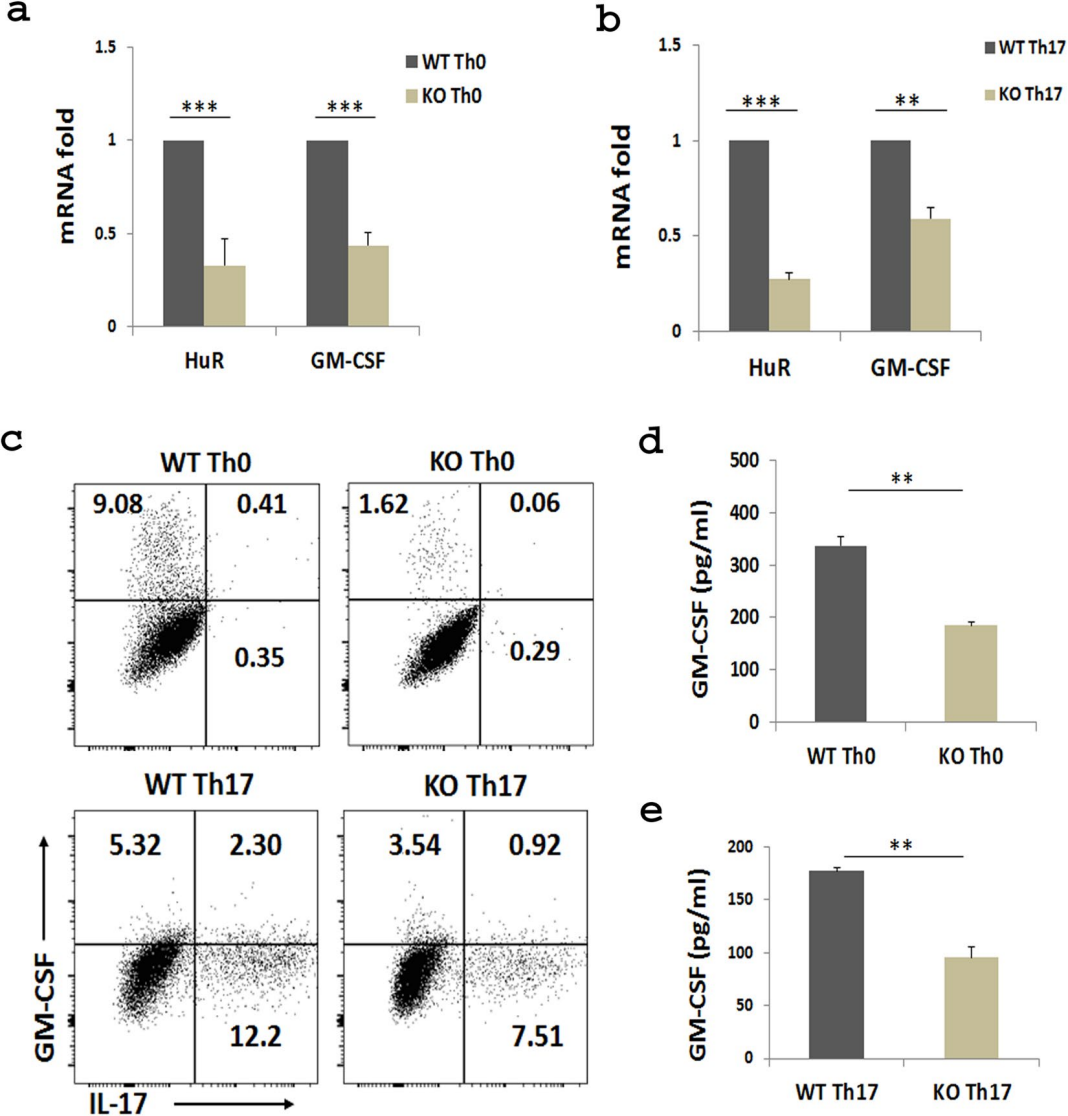
Knockout of HuR reduces GM-CSF mRNA in CD4^+^ T cells. Naive WT and HuR KO CD4^+^ T cells were isolated as described in Materials and Methods and cultured with anti-CD3 and anti-CD28 stimulation in Th0 and Th17 cell polarizing conditions. Expression of GM-CSF mRNA (**a**,**b**) and protein (**c–e**) in Th0 and Th17 cells was measured by qRT-PCR, flow cytometry, and ELISA, respectively. Knockout of HuR reduced the GM-CSF mRNA and protein levels in Th0 and Th17 cells compared with WT counterparts. Results in (**a**,**b**,**d** and **e**) represent the summary of three independent experiments. The representative of flow analysis was shown in (**c**). **p < 0.01, ***p < 0.001.

Our previous studies showed that the levels of HuR mRNA and protein correlate with IL-17 mRNA and protein expression in Th17 cells and that knockout of HuR reduces IL-17 mRNA and protein levels in Th17 cells^29^. Considering that GM-CSF plays a non-redundant role in Th17 cell induction of autoimmune demyelination^7,14^, we were interested in determining if HuR regulates GM-CSF mRNA expression in Th17 cells. To address this question, we used HuR conditional KO (OX40-Cre HuR^f/f^) mice^29^. Naive CD4^+^ T cells do not express OX40, but activated T cells do^36^, thus deleting HuR. An advantage of using this approach is that conditional knockout of HuR does not affect early development of T cells. Naive CD4^+^ T cells were isolated from wild type (WT) and KO mouse splenocytes, then were cultured under Th0 and Th17 cells-polarizing conditions. Western blot assays were performed to quantify HuR levels. Endogenous HuR was gradually degraded after cell activation in KO CD4^+^ T cells from day 3 to 5 of culture, and HuR was barely detected at day 5^29^. Quantitative real-time PCR (qRT-PCR) data showed that KO Th0 and Th17 cells had significantly less GM-CSF mRNA as compared with WT counterparts on day 4 of culture (Fig. 1a,b). Flow cytometry analysis also showed that the GM-CSF protein was also reduced in HuR KO Th0 and Th17 cells in comparison with WT cells (Fig. 1c). Results were confirmed by ELISA assay (Fig. 1d,e). These results demonstrated that knockout of HuR reduced GM-CSF mRNA and protein in Th0 and Th17 cells.

### HuR binds to GM-CSF mRNA 3′UTR

**Figure 2:**
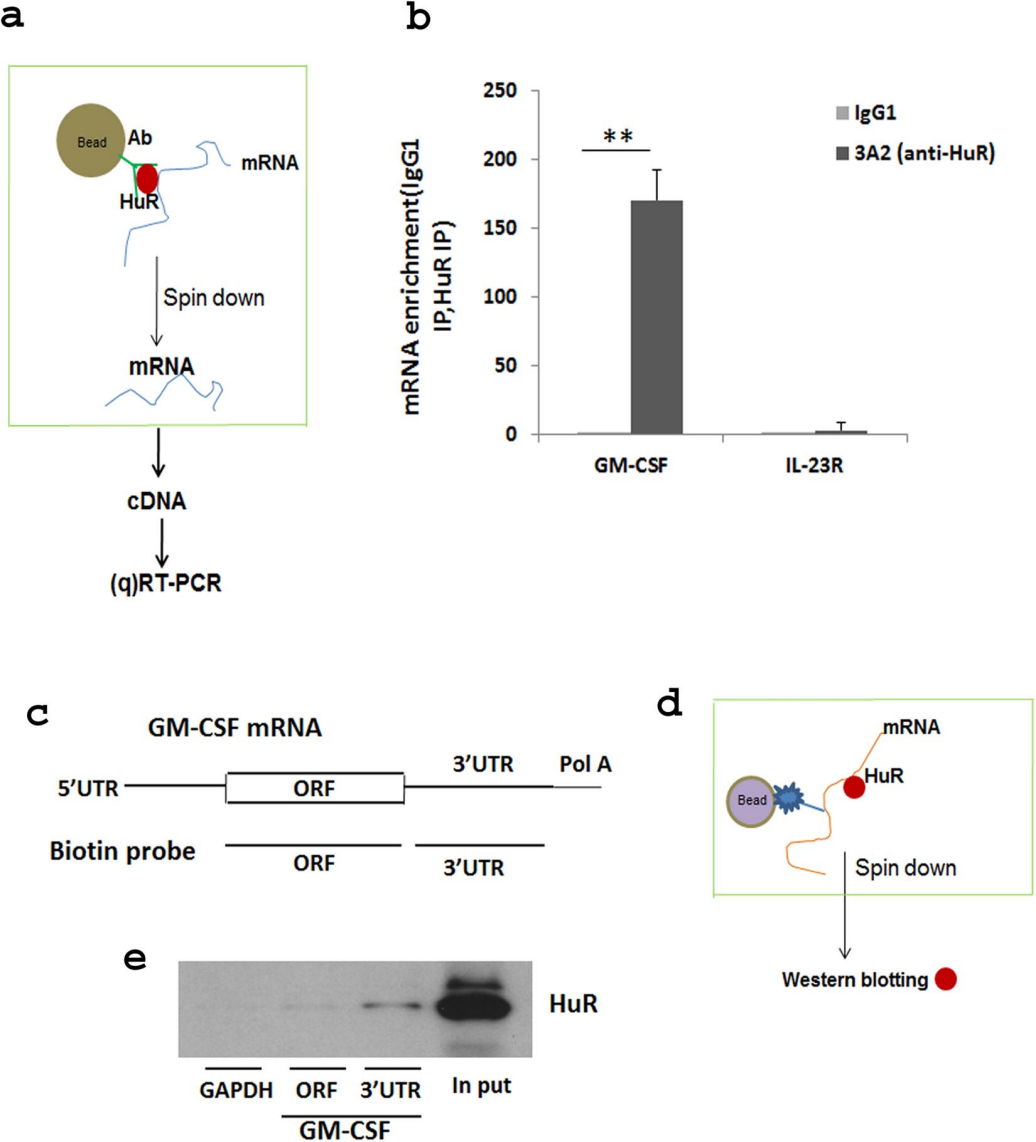
HuR regulates GM-CSF mRNA by binding and stabilizing its 3′UTR in Th17 cells. (**a**) Schematic diagram of RIP analysis. (**b**) RIP analysis was performed to detect GM-CSF mRNA enrichment after IP using anti-HuR (3A2) or isotype–matched antibody (IgG1) from Th17 cell cytoplasmic extracts. IL-23R mRNA was assayed as a negative control. (**c–e**) Biotin pulldown was used to determine whether HuR directly binds to 3′UTR of GM-CSF mRNA. (**c**,**d**) Schematic diagrams of biotin probe corresponding to GM-CSF and 3′UTR and biotin-pull down assay, respectively. (**e**) HuR protein could be detected by Western blot in biotinylated transcripts spanning GM-CSF 3′UTR, but not in GM-CSF mRNA ORF biotinylated transcripts. GAPDH biotinylated transcripts were unreactive (**e**). One of two independent experiments is shown in (**e**). **p < 0.01.

The regulation of GM-CSF mRNA expression by HuR suggested that GM-CSF mRNA might be a direct target of HuR. To study the function of HuR in regulation of GM-CSF in Th17 cells, we did RNA immunoprecipitation (RIP) assay to test if HuR protein binds to GM-CSF mRNA. The association of GM-CSF mRNA with HuR was tested by isolating RNA from the ribonucleoprotein (RNP) complexes with anti-HuR Ab (Fig. 2a). Our results showed a remarkable GM-CSF mRNA enrichment in the anti-HuR immunoprecipitation (IP) sample compared with an isotype-matched IgG control (Fig. 2b). IL-23R mRNA was not enriched in the HuR pulldown sample as a negative control (Fig. 2b). To confirm HuR interaction with GM-CSF mRNA, we utilized biotin pulldown, a second independent method. Two biotinylated transcripts that are complementary to sequences in GM-CSF mRNA ORF (open reading frame) and 3′ UTR were generated according to a protocol described in Materials and Methods (Fig. 2c). The biotinylated transcripts were incubated with cytoplasmic protein lysates from NIH/3T3 cells, which contain large amounts of HuR protein. The result of western blot showed that HuR was clearly detected in the samples precipitated by the biotinylated transcript of GM-CSF mRNA 3′ UTR (Fig. 2c–e). HuR protein could not be detected in the GAPDH 3′ UTR biotinylated probe pulldown complex (Fig. 2e), and this transcript has no putative HuR binding sites^29^. Taken together, these results indicated that HuR directly binds to 3′ UTR of GM-CSF mRNA, suggesting a regulatory relationship. Thus, HuR binds to and stabilizes GM-CSF mRNA, resulting in increased mRNA accumulation in Th17 cells.

### HuR inhibits miR-466i expression in Th17 cells

**Figure 3:**
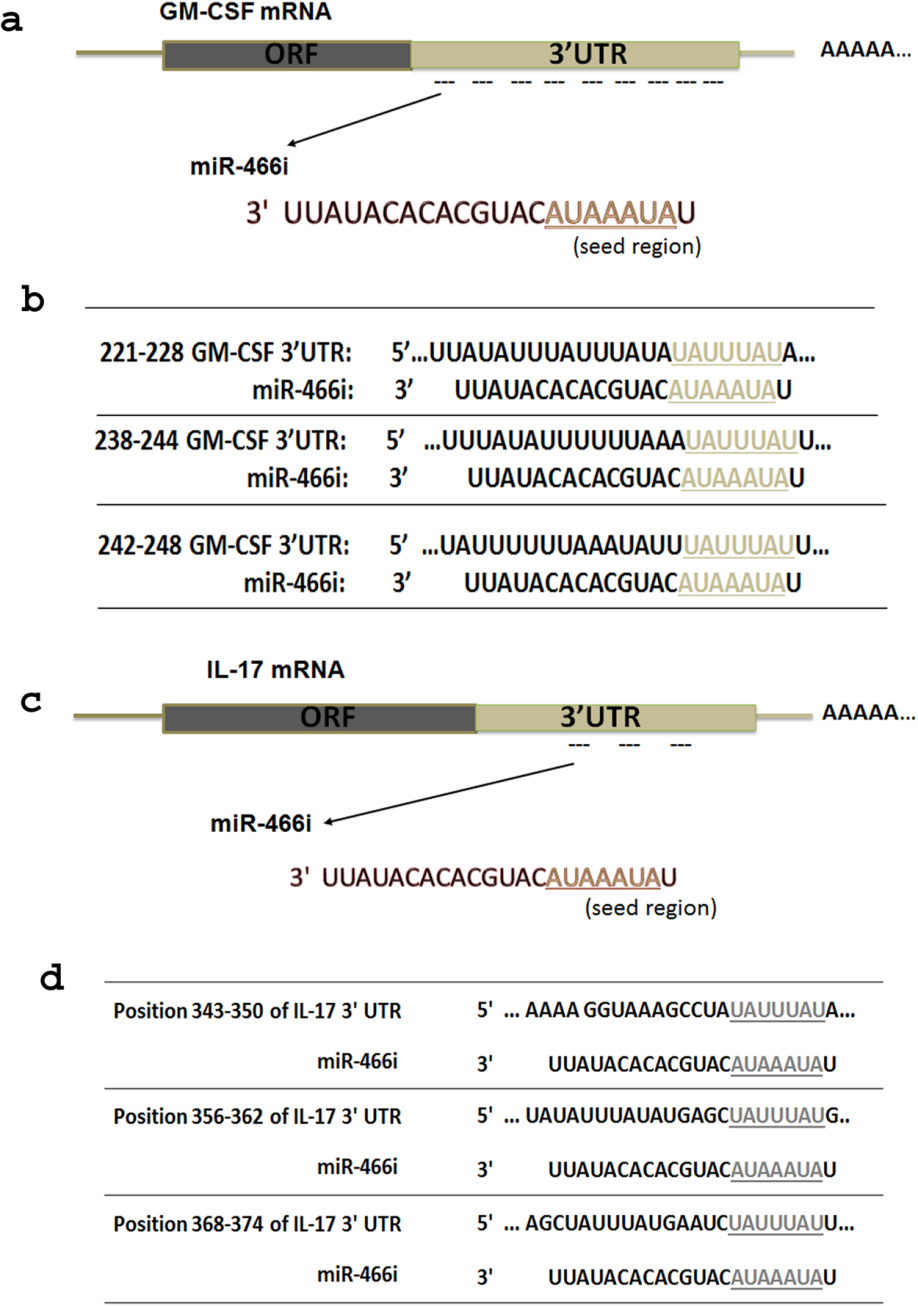
MiR-466i has multiple potential binding sites in GM-CSF and IL-17A mRNA 3′UTR. (**a**) There are nine miR-466i potential binding sites in 3′UTR of GM-CSF mRNA. There are three miR-466i potential binding sites listed in (**b**). Three miR-466i potential binding sites exist in 3′UTR of IL-17A mRNA which were listed in (**c**) and (**d**).

Since miRNAs play an important role in mediating target mRNA decay, decreasing mRNA levels by miRNAs represents a better approach to disrupt their protein function. We used several miRNA analysis programs to explore miRNAs that have the potential to target GM-CSF and IL-17 mRNAs. Interestingly, by Targetscan analysis, we found many miRNAs that could potentially bind to GM-CSF and IL-17 mRNAs, respectively (Supplementary Table 1). Among these miRNAs, it is only miR-466i that has potential binding sites in both GM-CSF and IL-17 mRNAs (Fig. 3a–d). Previous work showed that overexpression of miR-446i upregulates IL-10 expression in macrophages by antagonizing RNA-binding protein tristetraprolin-mediated IL-10 mRNA degradation, and miR-466i inhibits antiviral innate immune response by targeting interferon-α^37,38^. A recent study showed that miR-466i plays a bipolar role in inflammation, promoting an acute inflammatory response initially and enhancing resolution during the late stage of inflammation^39^. However, whether miR-466i mediates GM-CSF and IL-17 mRNA degradation remains unknown, thus, making this an important question to address.

**Figure 4:**
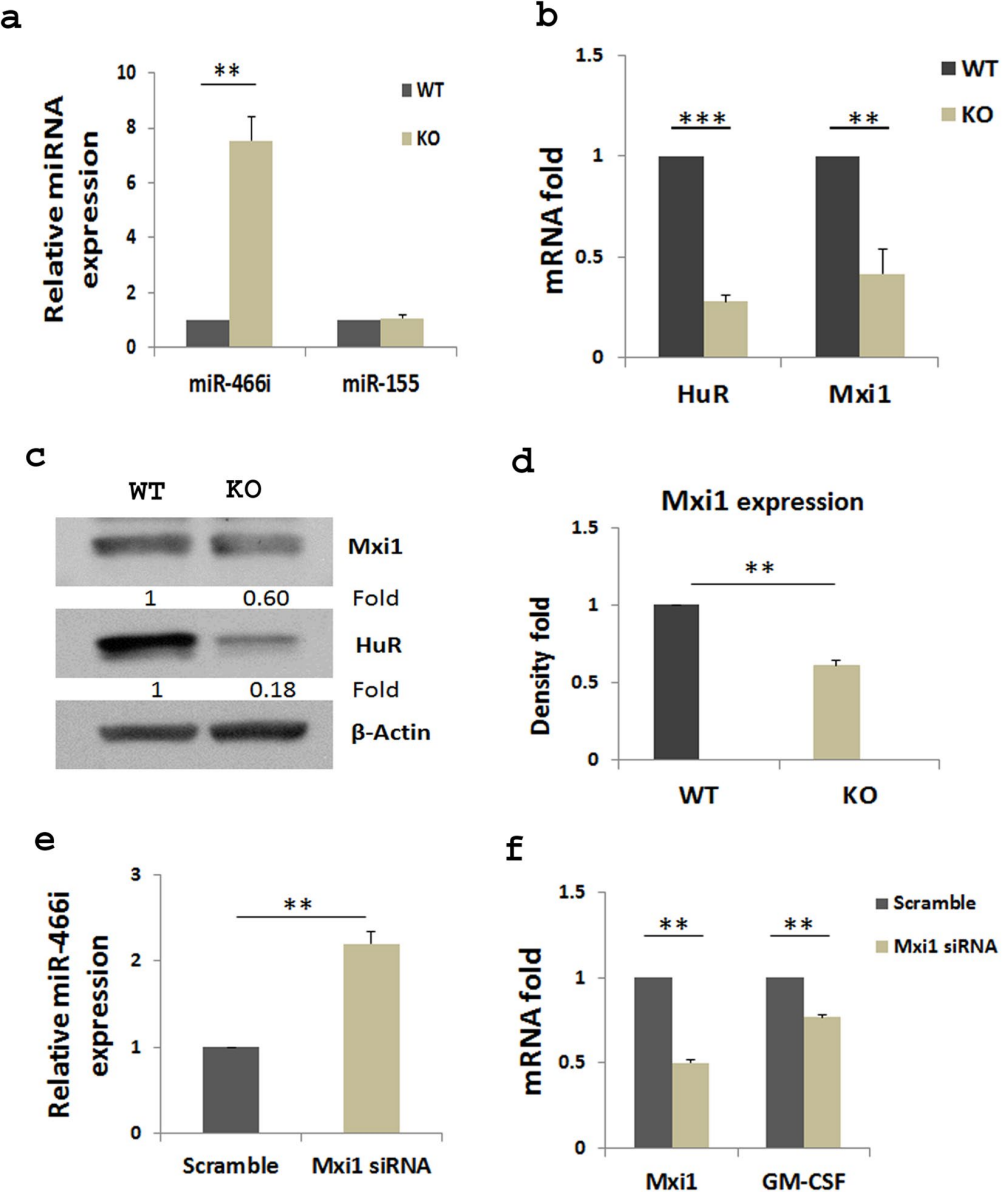
HuR inhibits miR466i but promotes Mxi1 expression in Th17 cells. Naïve CD4^+^ T cells were isolated from spleen of WT and HuR knockout mice and polarized as described in Materials and Methods. (**a**) Th17 cells were harvested after 5 days of cell culture. Expression of miRNAs in Th17 cells was analyzed by qRT-PCR and normalized by level of U6 snRNA. Expression of Mxi1 mRNA (**b**) and protein (**c** and **d**) was measured by qRT-PCR and Western blot, respectively. (**e**) Knockdown of Mxi1 in WT Th17 cells by Mxi1 siRNA transfection increased expression of miR-466i compared with the counterparts treated with scramble siRNA. (**f**) Knockdown of Mxi1 in WT Th17 cells also significantly reduced GM-CSF mRNA expression compared with WT Th17 cells transfected with scramble siRNA. Data in **a**, **b**, **d**, **e** and **f** summarize three individual experiments. The representative Western blots are shown in (**c**).

Interaction of RNA-binding protein HuR with miRNAs post-transcriptionally regulates gene expression^32^. Our previous studies showed that HuR post-transcriptionally modulates IL-17 mRNA expression and promotes autoimmune neuroinflammation^29^. Knockout of HuR reduces IL-17 mRNA half-life and destabilizes it. Here, we found that HuR directly regulated GM-CSF production by binding to 3′UTR of GM-CSF mRNA (Fig. 2b and e). We therefore performed qRT-PCR to examine miR-466i expression in WT and HuR KO Th17 cells. Interestingly, miR-466i was highly expressed in HuR KO Th17 cells in comparison with WT Th17 cells, but there was no significant change in miR-155 expression (Fig. 4a), suggesting that miR-466i may play an active role in mediating GM-CSF and IL-17 mRNA decay in the absence of HuR, and that HuR inhibits expression of certain miRNAs^27^.

To understand how HuR negatively regulates expression of certain miRNAs, we checked the expression of some molecules related to miRNA expression in HuR KO and WT Th17 cells. It is well known that Drosha, Dicer, Ago1/2 and c-Myc are actively involved in miRNA expression^40^, but their expression showed no significant change in the presence or absence of HuR (data not shown). However, expression of Mxi1 mRNA and protein, a repressor of c-Myc^41^, was significantly reduced in the absence of HuR compared with WT Th17 cells (Fig. 4b–d). Thus, it is possible that HuR promotes Mxi1 expression, which suppresses c-Myc function, resulting in its indirectly inhibiting expression of certain miRNAs. In line with this notion, knockdown of Mxi1 in WT Th17 cells by Mxi1 siRNA transfection increased miR-466i expression but decreased GM-CF mRNA compared with counterparts treated with scramble siRNA (Fig. 4e,f).

### MiR-466i-mediates GM-CSF and IL-17 mRNA decay by interacting with their 3′UTRs

**Figure 5:**
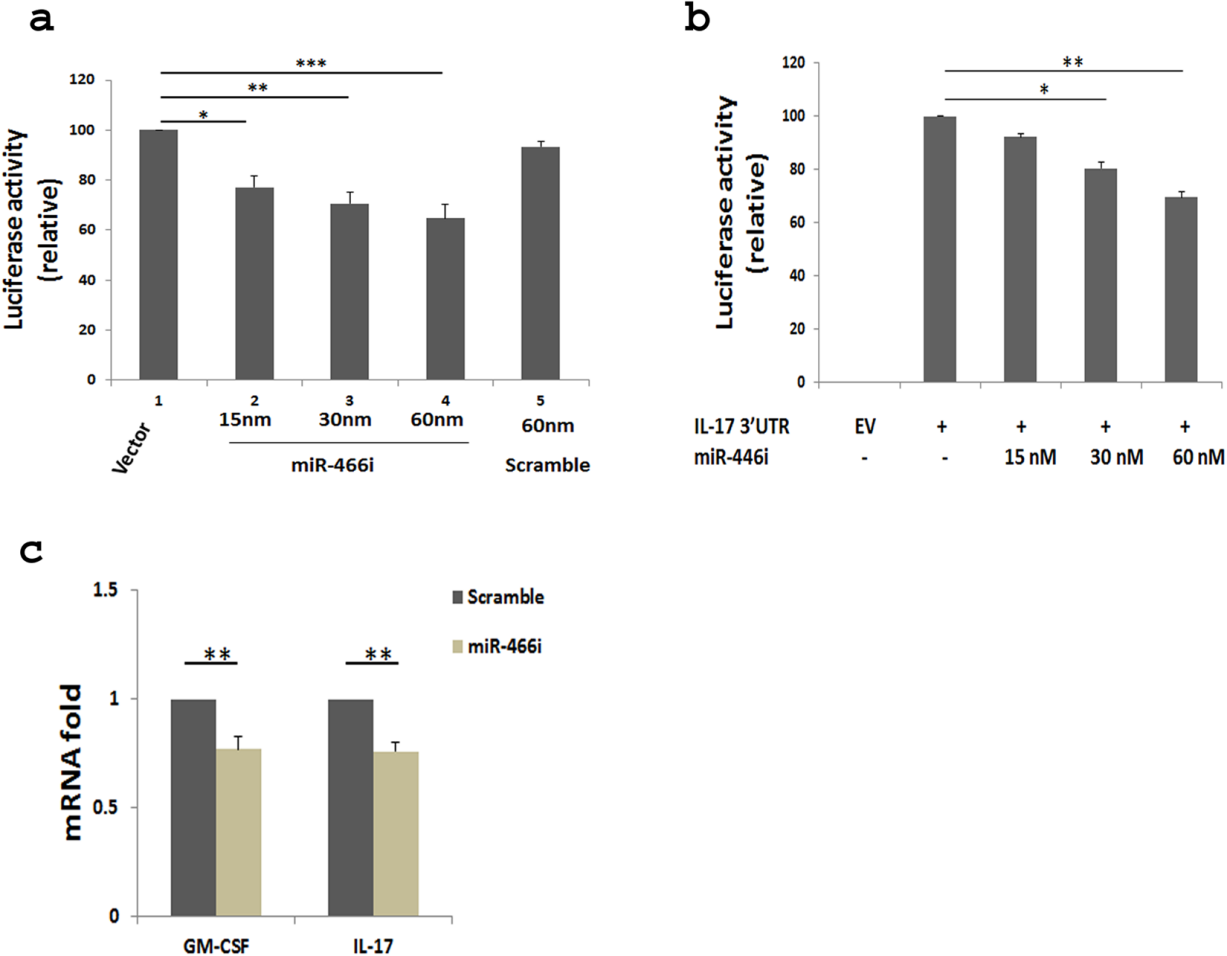
MiR-466i directly targets GM-CSF and IL-17 3′UTR. (**a**) HeLa cells were transfected with reporter containing GM-CSF 3′UTR, and overexpression of miR-466i decreased the luciferase activity of vector containing GM-CSF 3′UTR. (**b**) Similar results were obtained for the luciferase activity of vector containing IL-17 3′UTR. (**c**) Overexpression of miR-466i by transfection in Th17 cells reduced the expression of GM-CSF and IL-17 mRNA by qRT-PCR assay. Results in (**a–c**) are a summary of at least three independent experiments. EV (empty vector). *p < 0.05, **p < 0.01, ***p < 0.001.

To further investigate whether miR-466i modulates GM-CSF and IL-17 expression by degrading it through interacting with their 3′UTRs, a reporter vector with firefly and Renilla Dual-Luciferase (RDL) containing GM-CSF 3′UTR was co-transfected with miR-466i mimics into HeLa cells (Fig. 5a). Overexpression of miR-466i decreased luciferase activity of the reporter construct by analysis of firefly luciferase activity which was normalized by Renilla luciferase activity, but overexpression of scramble miRNAs did not (Fig. 5a), suggesting that miR-466i functions to degrade GM-CSF mRNA through binding to 3′UTR. Similarly, overexpression of miR-466i in HeLa cells transfected with RDL vector containing IL-17 mRNA 3′UTR also reduced luciferase activity (Fig. 5b). These results thus suggest that miR-466i mediates GM-CSF and IL-17 mRNA decay through binding to their 3′UTRs. In line with these results, overexpression of miR-466i in WT Th17 cells by transfection reduced expression of GM-CSF and IL-17 mRNA compared to that with scramble miRNA (Fig. 5c).

### MiR-466i regulates GM-CSF protein expression in macrophages

**Figure 6:**
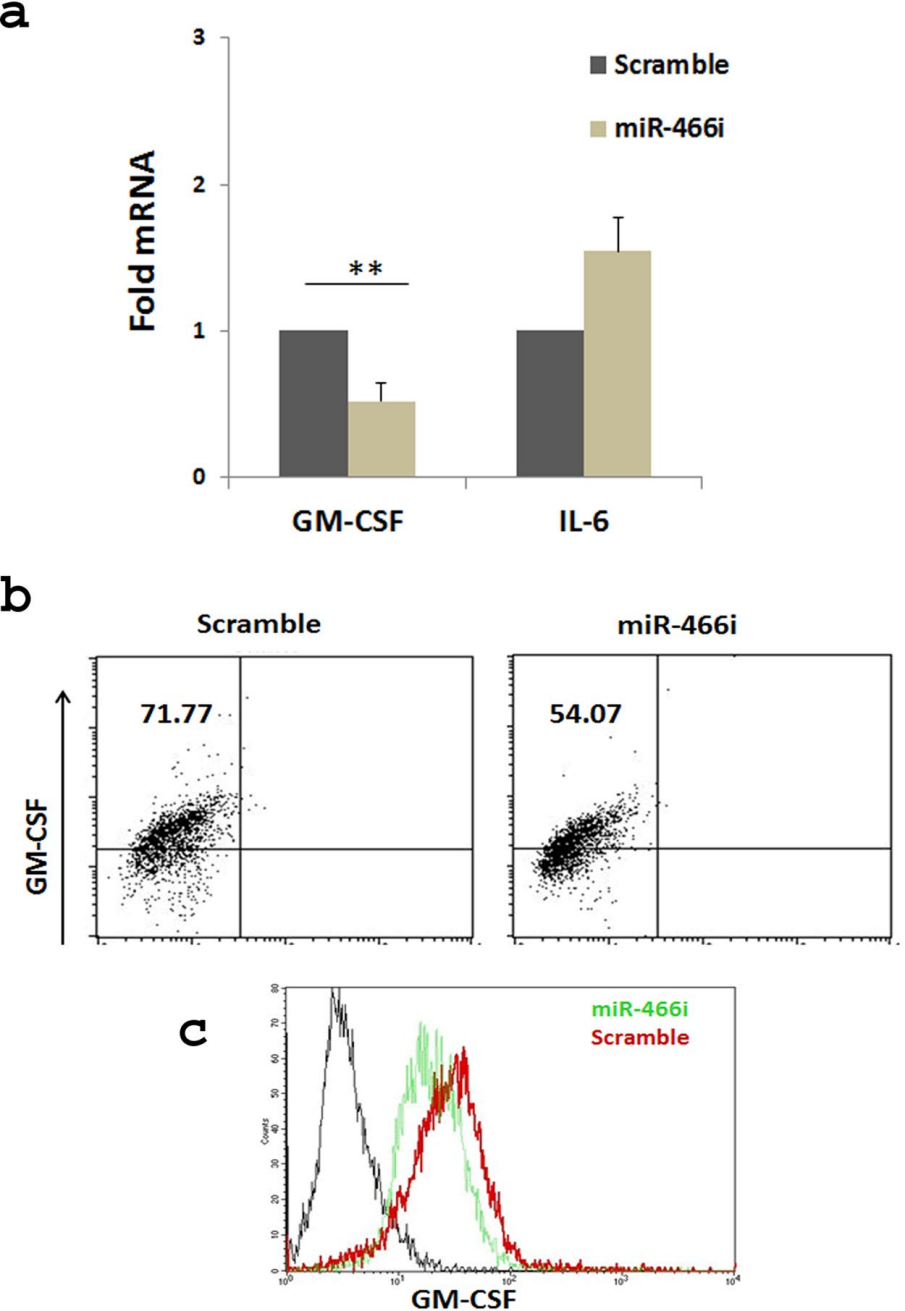
MiR-466i overexpression reduced GM-CSF expression in activated macrophages. RAW264.7 cells were transfected with miR-466i mimics or scramble control at a final concentration of 10 nM, and 24 hours later after LPS stimulation (100 ng/ml), the cells were harvested. (**a**) Expression of GM-CSF and IL-6 mRNA levels were measured by qRT-PCR analysis and normalized by GAPDH. **(b**,**c)** GM-CSF protein expression levels were measured by flow cytometry. Data in (**a**) are a summary of three individual experiments. The representative flow cytometric analysis is shown in (**b**) and (**c**).

To further confirm the result that miR-466i modulates GM-CSF expression, macrophages were transfected with miR-466i and scramble miRNAs. GM-CSF mRNA and protein levels were examined by qRT-PCR and flow cytometry, respectively. The data showed that expression of GM-CSF mRNA was remarkably decreased when macrophages were transfected with miR-466i compared with scramble miRNA (Fig. 6a); however, the IL-6 mRNA level was moderately increased in macrophages transfected with miR-466i, which is consistent with a previous report^39^. The GM-CSF protein level was also decreased in macrophages transfected with miR-466i mimics compared with scramble miRNA (Fig. 6b,c). This result indicated that miR-466i also regulates GM-CSF expression in macrophages.

Taken together, these results suggest that HuR and miR-466i interaction modulates GM-CSF expression in Th17 cells. MiR-466i targets GM-CSF and IL-17 mRNA decay by binding to their 3′UTRs, a finding that may lead to development of miR-466i as a novel therapeutic intervention for autoimmune inflammation.

## Discussion

MiRNAs are small (approximately 22 bases) non-protein-coding RNAs that recognize target sequences of imperfect complementarity in cognate mRNA and either destabilize them or inhibit protein translation. In principle, gene expression is post-transcriptionally regulated under the control of RNA-binding protein and miRNA interaction^32^. Our current study shows that HuR binds to GM-CSF and IL-17 mRNA by inhibiting miR-466i expression and by preventing miR-466i-mediated targeting of GM-CSF and IL-17 mRNA decay.

In this study, we first used OX40-Cre HuR^f/f^ mice to demonstrate that HuR promotes GM-CSF mRNA expression. Ablation of HuR reduced GM-CSF mRNA and protein expression in Th0 and Th17 cells. We previously reported that knockout of HuR impacted IL-17 expression^29^. GM-CSF and IL-17 in Th17 cells are thus similarly post-transcriptionally regulated by HuR.

Here we found that HuR functions to bind to GM-CSF mRNA 3′UTR. HuR ablation therefore reduced GM-CSF mRNA expression, which is consistent with previous reports showing that GM-CSF can be controlled by a post-transcriptional mechanism through ARE in its 3′UTR and stabilized by HuR^42–45^. However, the mechanism by which GM-CSF mRNA was degraded in the absence of HuR had not been completely characterized in previous reports. Thus, our current study extended previous findings by showing that HuR deficiency in T cells using conditional knockout mice deceased GM-CSF production and increased miR-466i expression. *In vitro* analysis suggested that miR-466i has the capacity to mediate GM-CSF and IL-17 mRNA decay. The fact that knockout of HuR increased miR-466i, miR-409 and miR-335 expression^30^ is consistent with a previous report showing that overexpression of HuR reduces miR-16 expression, and that HuR inhibits miR-16 targeting of Cox-2^46^. To understand why knockout of HuR increased certain miRNA expression, we also performed anti-HuR immunoprecipitation (IP) western blots to determine whether HuR and Ago2 are physically associated with each other. Not surprisingly, Ago2 could not be detected in the anti-HuR IP complex (data not shown). It has been reported that c-Myc is a transcription factor with the capacity to promote certain miRNA expression^47,48^, and that HuR recruits miRNA let-7 to mediate c-Myc RNA decay^49^. Mxi1 is a transcription repressor that negatively regulates c-Myc function^41^. Although knockout of HuR did not impact the expression of c-Myc mRNA (data not shown), Mxi1 mRNA and protein expression was significantly reduced in the absence of HuR (Fig. 4). Therefore, knockout of HuR reduced Mxi1 expression, which may alleviate its repressive effect on the function of c-Myc, leading to an increase in expression of certain miRNAs. Based on this idea, we speculated that knockdown of Mxi1 in WT Th17 cells would result in increased miR-466i expression. Interestingly, we obtained new data which support this notion (Fig. 4). However, how HuR modulates miRNA biogenesis via Mxi1 only and/or other factors need to be further studied.

By *in vitro* luciferase assay, we demonstrated that overexpression of miR-466i reduced activity of the vector containing GM-CSF or IL-17 mRNA 3′UTR in transfected Hela cells, suggesting that miR-466i has the potential to target GM-CSF and IL-17 mRNA. Indeed, overexpression of miR-466i in WT Th17 cells and macrophages decreased GM-CSF mRNA and its protein production, respectively (Fig. 6). A previous report showed that miR-466i increased anti-inflammatory cytokine IL-10 production by blocking RNA-binding protein tristetraprolin, which destabilized IL-10 mRNA^37^. A recent report demonstrated that miR-466i could target MyD88 mRNA 3′UTR for decay to downregulate IL-12 production and increase IL-10 production^50^. miR-466i might therefore have the therapeutic potential to target GM-CSF-mediated inflammation and to increase IL-10 production for promoting resolution of inflammation.

Since HuR binds to GM-CSF 3′UTR (Fig. 2), and GM-CSF 3′UTR contains nine potential miR-466i binding sites (Supplementary Table 1), it is likely that HuR and miR-466i might competitively bind to the same target^32^. However, we cannot rule out the possibility that with HuR binding, the space-structure of GM-CSF mRNA 3′UTR changes, which prevents miR-466i from binding to it^32^. In any case, HuR inhibition of miR-466i biogenesis also contributes to the effect of HuR on promoting GM-CSF expression.

Overall, RNA-binding protein HuR post-transcriptionally regulates GM-CSF and IL-17 mRNA expression by binding to target mRNAs. HuR promotes Mxi1 expression to inhibit the expression of certain miRNAs. HuR and miR-466i interaction orchestrates GM-CSF and IL-17 mRNA fate. Taking advantage of the many newly developed small molecule inhibitors for HuR in anti-tumor research^51,52^, and based on our current study on HuR regulation of GM-CSF production, it would be worthwhile to test the effects of these newly developed HuR inhibitors on anti-autoimmune inflammation.

## Materials and Methods

### Cell culture

Mouse naive CD4^+^ T cells were isolated from splenocytes of WT and HuR conditional knockout (HuR KO) mice^29^ using CD4 negative selection kits (StemCell Tech., Vancouver, Canada) following the manufacturer’s protocol. Cells were differentiated into Th17 cells as previously described^29,53^. Isolated naive CD4^+^ T cells were activated with plate-bound anti-CD3 (10 µg/ml) and soluble anti-CD28 (3 µg/ml) in the presence of TGF-β (3 ng/ml), IL-6 (20 ng/ml), IL-23 (20 ng/ml), anti-IFN-γ (10 µg/ml) and anti-IL-4 (10 µg/ml). Naïve CD4+ T cells stimulated with anti-CD3 and anti-CD28 and without other polarizing cytokines were designated as Th0 cells. All cytokines were purchased from R&D Systems (Minneapolis, MN) and Peprotech (Rocky Hill, NJ), and antibodies were purchased from eBiosciences (San Diego, CA) and Biolegend (San Diego, CA).

RAW 264.7 macrophages (ATCC) were cultured in DMEM with 10% FBS plus penicillin (100 U/ml) and streptomycin (100 μg/ml) in 5% CO_2_ atmosphere at 37 °C.

All mice used are on the C57BL/6 background and were breed at the animal facility of Arkansas Bioscience Institute at Arkansas State University and/or the Thomas Jefferson University. Animal experiments were approved by the Arkansas State University (ASTATE) and Thomas Jefferson University (TJU) IACUC committee. We followed the regulation of NIH, ASTATE and TJU according to federal and institutional guidelines.

### Flow cytometry

Cells obtained from *in vitro* 5 day culture were stained for surface markers with FITC-conjugated anti-CD4, PE-conjugated anti-GM-CSF, and allophycocyanin-conjugated anti-IL-17 (Biolegend, San Diego, CA). Acquisitions were made with a BD FACSAria II (BD Biosciences, San Diego, CA). Flowjo software was used for data analysis.

### RNA isolation and Quantitative real time-PCR (qRT-PCR)

Cultured cells were collected and total RNA was extracted using TRIzol (Invitrogen, Life Technologies Corp., Thermo Fisher Scientific). Five hundred ng of RNA was reverse transcribed into cDNA using SuperScript III Kit (Life Technologies, Fisher Thermos Scientific Inc.) according to the manufacturer’s protocols. The cDNA was subjected to qRT-PCR analysis using the CFX96 Real-Time PCR Detection system (Bio-Rad, Hercules, CA) with SYBR Green reagent Kit (Invitrogen, Life Technologies Corp., Thermo Fisher Scientific) according to the manufacturer’s protocols. The levels of test mRNAs were normalized to the levels of *Gapdh* mRNA for each sample. Forward and reverse primers for specific murine target genes are listed in Table 1.

**Table 1:** Sequences of mouse quantitative RT-PCR primers.

HuR forward	5′-ACTGCA GGGATGACATTGGGAGAA-3′
HuR reverse	5′-AAGCTTTGCAGATTCAACCTCGCC-3′
GM-CSF forward	5′-TGGAAGCATGTAGAGGCCATCA-3′
GM-CSF reverse	5′-GCGCCCTTGAGTTTGGTGAAAT-3′
IL-23R forward	5′-TTCAGATGGGCATGAATGTTTCT-3′
IL-23R reverse	5′-CCAAATCCGAGCTGTTGTTCTAT-3′
Mxi1 forward	5′-AACATGGCTACGCCTCATCG-3′
Mxi1 reverse	5′-CGGTTCTTTTCCAACTCATTGTG-3′
IL-6 forward	5′-CTTCACAAGTCGGAGGCTTAAT-3′
IL-6 reverse	5′-GCAAGTGCATCATCGTTGTTC-3′
GAPDH forward	5′-TCAACAGCAACTCCCACTCTTCCA-3′
GAPDH reverse	5′-ACCCTGTTGCTGTAGCCGTATTCA-3′

### RNA Immunoprecipitation (RIP)

RIP was performed according to published protocols^29,54^. Th17 polarized cells at day 4 to 5 culture were lysed using polysome lysis buffer supplemented with RNase inhibitors and protease inhibitors^55^. Lysates were pre-cleared by adding 30 µg of IgG_1_ (BD Bioscience) and 50 µl of Protein-A/G Sepharose beads swollen in NT2 buffer with 5% BSA. Beads were coated by adding either IgG1 (BD Biosciences, San Diego, CA) as control or anti-HuR antibody 3A2, and incubated overnight at 4 °C. After extensive washes of pre-coated Protein-A/G sepharose beads, the pre-cleared lysate was added and incubated for 4 h at 4 °C, and then 30 µg of proteinase K was added to digest protein by incubation at 55 °C for 30 min. The extracted RNA was reverse transcribed into cDNA, and qRT-PCR was preformed to detect the presence of specific target mRNAs.

### Biotin pulldown assay

Biotinylated transcripts were synthesized as previously described^29^. Forward primers that contained the T7 RNA polymerase promoter sequence (5′-CCAAGCTTCTAATACGACTCACTATAGGGAGA-3′ [T7]) and reverse primers used to generate cDNA are listed as following: GM-CSF ORF forward plus T7 5′-GCTTCTAATACGACTCACTATAGGATGTGGCTGCAGAA-3′; GM-CSF ORF Reverse: 5′-TCATTTTTGGCCTGGTTTTTTGCATTCAAAGGGG-3′; GM-CSF 3′UTR Forward plus T7: 5′-GCT TCTAATACGACTCACTATAGGGGAAGCCCAGGCCAG-3′; GM-CSF 3′UTR Reverse: 5′-CTG GTAAGACATTCTCAATAAATAGA-3′. PCR-amplified products were purified and used as templates for the synthesis of biotinylated RNA with T7 RNA polymerase and biotin-conjugated UTP for murine RNAs. Biotinylated transcripts were incubated with NIH/3T3 cell lysate at room temperature. The RIP complexes were isolated with paramagnetic streptavidin-conjugated Dynabeads (Invitrogen, Life Technologies Corp., Thermo Fisher Scientific). Bound HuR protein in the pulldown pellet was analyzed by western blots to evaluate whether HuR directly binds to 3′ UTR of GM-CSF mRNA.

### Transfection and luciferase assay

HeLa cells (ATCC) were cultured in DMEM containing 10% FBS, penicillin (100U/ml), streptomycin (100 μg/ml), 2 mM L-glutamine and non-essential amino acids (Invitrogen, Thermo Fisher Scientific) in 24-well plates and incubated overnight. Lipofectamine 2000 (Invitrogen, Life Technologies Corp., Thermo Fisher Scientific) was used to transfect Hela cells with pRL-CMV-renilla-luciferase plasmid and firefly-luciferase plasmid DNA containing GM-CSF or IL-17 mRNA 3′UTR. Hela cells were co-transfected with 40–80 nmol miR-466i and scramble miRNAs (Life Technologies, Thermo Fisher Scientific). Twenty-four hours later, cells were lysed and luciferase activities were measured using a Dual-Luciferase Reporter Assay System according to the manufacturer’s instructions (Promega, Madison, WI). Firefly luciferase activity was normalized to renilla luciferase in each sample.

For transfection of siRNA, naïve WT CD4^+^ T cells were transfected with Mxi1 siRNA and scramble siRNA (Life Technologies Corp., Thermo Fisher Scientific) using Lonza 4D-nucleofector according to the manufacturer’s instructions.

### Statistics

Bar graphs in figures represent average values ± SEM unless indicated otherwise. Statistical significance between experimental groups was calculated using a two-tailed unpaired Student *t* test and is shown in the graphs as follows: *p < 0.05, **p < 0.01, ***p < 0.001.

## Electronic supplementary material

Supplementary Table 1
