# Supplementary material for: Interaction of RNA-binding protein HuR and miR-466i regulates GM-CSF expression
Source: Sci Rep. 2017 Dec 8;7:17233. doi: 10.1038/s41598-017-17371-5 (PMC5722853; doi:10.1038/s41598-017-17371-5)
Supplement: Supplementary file 1 — Supplementary Table 1 [file 41598_2017_17371_MOESM1_ESM.doc]

**Interaction of RNA-binding protein HuR and miR-466i regulates GM-CSF expression**

Jing Chen^1, 2, *^, William Adamiak^1^, Ganlei Huang^2^, Ulus Atasoy^3^, Abdolmohamad Rostami^1^ and Shiguang Yu^1, 2,*^

1 Department of Neurology, Thomas Jefferson University, Philadelphia, PA 19107

2 Arkansas Biosciences Institute, Arkansas State University, Jonesboro, AR 72467

3 Department of Molecular Microbiology and Immunology, Department of Surgery, University of Missouri, Columbia, MO 65211

**Running title**: HuR and miRNA regulate GM-CSF expression

***Correspondence**: Dr. Jing Chen or Dr. Shiguang Yu, Department of Neurology, Thomas Jefferson University, 900 Walnut St, JHN 3^rd^ Floor, RM 300, Philadelphia, PA 19107.

Email: [Jing.chen@jefferson.edu](mailto:Jing.chen@jefferson.edu) or [Shiguang.yu@jefferson.edu](mailto:Shiguang.yu@jefferson.edu); Tel: 215-955-0901


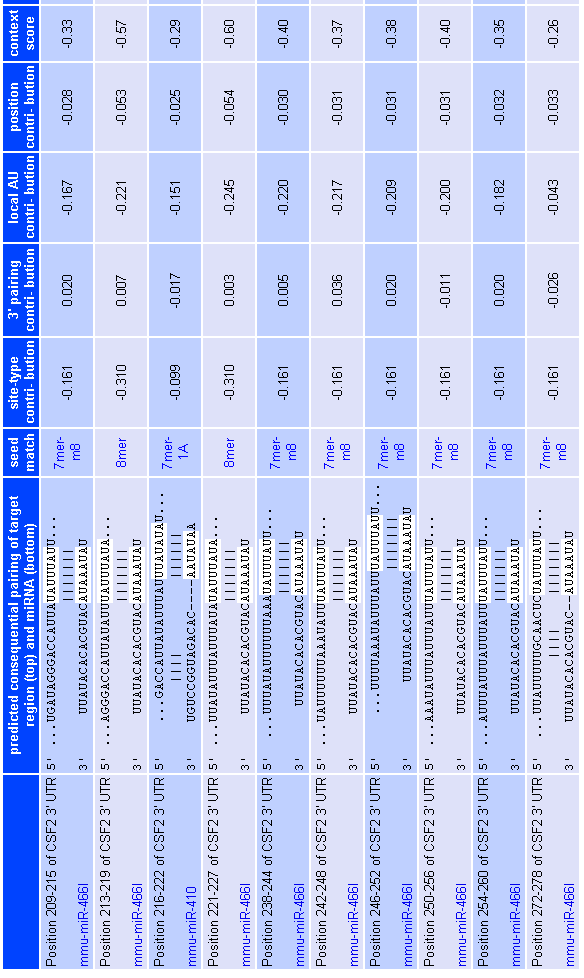


**Supl. Table 1. Targetscan analysis for predication of miRNAs which could potentially target GM-CSF mRNA**
